# The Cellulose Loading and Silylation Effects on the Mechanical Properties of Epoxy Composites: Insights from Classical and Reactive Molecular Dynamics Simulations

**DOI:** 10.3390/polym17202749

**Published:** 2025-10-14

**Authors:** Ahmad Y. Al-Maharma, Bernd Markert, Franz Bamer

**Affiliations:** Institute of General Mechanics, RWTH Aachen University, Eilfschornsteinstraße 18, 52062 Aachen, Germany; almaharma@iam.rwth-aachen.de (A.Y.A.-M.); markert@iam.rwth-aachen.de (B.M.)

**Keywords:** cellulose, epoxy, silylation, strength, elasticity, ReaxFF

## Abstract

This study investigates the effect of silylation and cellulose loading on the mechanical properties of epoxy composites. We use the hydrolyzed 3-Aminopropyltriethoxysilane (KH550) as a crosslinker for epoxy and as a coupling agent for cellulose. The mechanical properties of the epoxy composites are evaluated using molecular dynamics simulations. The improvement in the interfacial adhesion between epoxy and cellulose, achieved by using KH550, is demonstrated through the pulling out of cellulose from the epoxy composites. The results indicate that the nanocovalent bonds formed by KH550 at the epoxy/cellulose interface have a higher enhancement effect on the pulling force compared to increasing the cellulose content. For instance, the force needed for pulling 44.1 wt.% of raw cellulose is 93 ± 5 (kcal/mol)/Å, while the one required to pull the 28.1 wt.% of silylated cellulose is 97 ± 4 (kcal/mol)/Å. The silylated cellulose at 28.1 wt.% enhances the tensile modulus, shear modulus, and strength of the epoxy-KH550 composite by 14.55%, 15.65%, and 15.64%, respectively, compared to its counterpart reinforced with raw cellulose. Using the silylation treatment on cellulose that reinforces epoxy-KH550 at 43.9 wt.% improves the elastic modulus, shear modulus, and tensile strength of the epoxy composite by 4.23%, 4.64%, and 18.07%, respectively.

## 1. Introduction

Cellulose exhibits attractive ecological properties, characterized by high stiffness and strength at a low weight [1]. The material properties of cellulose-derived materials, such as wood, are highly influenced by their density. The densification improves the homogeneity of the material and its absolute mechanical properties. Regenerated cellulose differs significantly from plant fibers regarding its properties and structure. Fibers are partially crystalline (cellulose II) and highly aligned, with the degree of alignment higher in the external surface relative to the core of the fiber. The highly aligned cellulose nanofibrils (CNFs) exhibit an ultimate tensile strength of 576 ± 54 MPa and stiffness of 32.3 ± 5.7 GPa. The fibers composed of regenerated cellulose are highly extensible, with a strain to failure between 8.0% and 25% relative to natural fibers, which ideally fail at 1.0–2.0% strain, because of the relatively high percentage of non-crystalline cellulose [2,3]. The fibers synthesized from regenerated cellulose exhibit good performance in terms of work to fracture, making them a suitable option for industrial applications where high fracture toughness is one of the requirements [4]. For instance, the regenerated cellulose fiber of lyocell has an ultimate tensile strength of 556 ± 78 MPa and a failure strain of 8.7 ± 1.6% [5].

Cellulose-derived reinforcements significantly increase the stiffness, tensile strength, fracture resistance, elongation at break, and impact energy absorption of epoxy composites [6,7,8,9]. The CNF at a 0.1 weight fraction (wt.%) increases the tensile strength of epoxy by 6.45% [10]. The stiffness, strength, and fracture toughness of epoxy composites can be significantly improved by reducing the porosity at the epoxy/cellulose interface and increasing the aspect ratio and content of cellulose reinforcements [11]. The cellulose reinforcements at high contents have better interfacial bonding with epoxy if they have homogeneous dispersion in the hosting thermoset [12]. The long cellulose filaments improve the flexural strength and modulus of bio-based vanillin epoxy significantly by 542.8% and 135.1%, respectively [13]. Increasing the content of microcrystalline cellulose (MCC) increases the ultimate strength, failure strain, and stiffness of the epoxy thermoset. The particles of MCC arrest the cracks propagating within the epoxy, which improves the fracture energy of the epoxy composites [14]. Incorporating 18–23 wt.% of CNF into epoxy nanocomposites noticeably increases their mechanical properties [15]. Reinforcing epoxy with CNF at 20–36 wt.% doubles the tensile modulus and strength of the resulting composites [16]. Adding 13% volume fraction (vol.%) of CNF to bioepoxy increases the ultimate strength by 20.2% and storage modulus by 50% [17].

The cellulose nanofillers, such as CNF, can be utilized to mitigate the adverse impact of moisture on the epoxy nanocomposites. CNF can be blended with graphene oxide to develop a high-humidity-sensing filament. The capability of detecting humidity of the developed filament can be observed by the resistivity changes in different humidity and temperature levels [18]. The presence of a high aspect ratio and high crystalline CNF at 23 wt.% mitigates the moisture permeability of the pure epoxy thermoset with a percentage exceeding 50% [15]. The surface of regenerated cellulose fiber is highly polar since it is rich with hydroxyl groups that interact with the surrounding atmosphere, enabling the fiber to adsorb water. The interfacial adhesion between epoxy and cellulose reinforcements is considerably degraded when the fibers absorb moisture from a humid environment, causing a reduction in the material aspects of the epoxy/cellulose composite. The cellulose fiber absorbs moisture, which in turn reduces the fracture toughness, flexural modulus, and strength of the epoxy composite. The use of an appropriate coupling agent is necessary to enhance the interfacial properties between regenerated cellulose fibers and the non-polar thermoset of epoxy. The cellulose reinforcements can be chemically modified using their hydroxyl groups, enabling different methods for surface chemical modification [2,7,19].

The hydrolyzed (3-aminopropyl)triethoxysilane (APTES), known as KH550, is one of the most essential silane coupling agents used for cellulose functionalization [20]. Three different types of silane agents, KH550, KH792, and KH570, are used to chemically functionalize the interface between epoxy and glass fiber. These silane coupling agents are ranked in terms of their effectiveness in improving the tensile mechanical properties, including tensile strength, in the following order: KH550 > KH792 > KH570 [21]. The surface of cellulose nanofillers, such as cellulose nanocrystal (CNC), can be silylated firstly by hydrolyzing APTES in water and then adsorbing it onto CNC via the hydrogen bonds; eventually, the surface of CNC has covalent bonds with the chain hydrocarbon through the Si-O-C bonds, which are generated by the condensation reaction among silanol groups and hydroxyl [22,23]. Adding 1.0 wt.% of silylated CNF into poly(methyl methacrylate) (PMMA) resin improves its tensile strength by more than two times [24]. The silylation treatment enhances the tensile strength and failure strain of bamboo fiber-reinforced epoxy composite by 71% and 53%, respectively. The enhancements attained on the mechanical properties of epoxy composites reinforced with silylated cellulose can be justified by the generation of interfacial covalent bonds linking cellulose with epoxy, thereby enhancing their interfacial interactions [25]. Incorporating 3 wt.% of APTES into the epoxy thermoset improves the failure strain and tensile strength of epoxy composite reinforced with viscose fabric by 14% and 41%, respectively. The enhancement in the tensile properties is justified by the increased interfacial interactions between epoxy and viscose fabric after the resin modification [26]. Modifying the epoxy thermoset with 5 wt.% of APTES increases the toughness of the epoxy composite reinforced with viscose fabric by twofold. The functionalization of the epoxy thermoset by APTES is considered a suitable option when taking into account low energy consumption and less process waste relative to wet chemical functionalization methods such as acetylation and alkali treatments [27].

Molecular-based methods, such as molecular mechanics and molecular dynamics (MD) simulations, are extensively used in research to investigate the relationship between the material’s structure and its mechanical properties [28,29]. Introducing defects into a material’s structure or altering the interfacial adhesion between its components significantly affects its mechanical properties. The effect of these changes on the mechanical properties cannot be quantitatively determined using experimental setups. The MD simulations can be utilized to evaluate the interfacial properties among surfaces synthesized from the same and different materials. For instance, the interfacial adhesion among the surfaces of metallic welds can be effectively characterized using MD simulations [30]. Additionally, molecular simulation is employed to investigate the impact of chemical functionalization on the durability and the interfacial behavior of cellulose nanofiber-reinforced epoxy nanocomposites under salt and aqueous environments. The cellulose chain is functionalized by adding carboxyl groups to the external surface of cellulose nanofiber [31]. However, the reactive MD simulation has not been implemented yet to evaluate the impact of silylation treatment on the mechanical properties of epoxy composites containing cellulose reinforcements. Additionally, previously published research has not yet addressed the effectiveness of the silylation treatment in improving the mechanical properties of epoxy composites at high weight fractions of cellulose. To the best of our knowledge, we are among the leading scholars who implemented the MD simulations in exploring the effect of the silane coupling agent KH550 in improving the interfacial adhesion between epoxy and cellulose through the interfacial nanocovalent bonds. The results of our MD simulations conducted using classical and reactive force fields are summarized and presented via a preprint posted online [32]. The current study is developed based on the aforementioned preprint to characterize the relationship between the stress transferred through the epoxy/cellulose interface by the nanocovalent bonds and the content of cellulose reinforcement in the epoxy composite. The recent studies, which were published after our preprint [32], depend on the MD simulation to investigate the properties of interfacial adhesion in composite materials containing either cellulose or epoxy in their compositions. Wang et al. [33] used the classical force field of CHARMM to predict the binding energy between functionalized cellulose and polypropylene (PP) depending solely on the non-bonded interactions of hydrogen atoms between them. The results of their study indicated that the chains of raw cellulose have better binding energy with PP relative to their chemically treated counterparts. If a layer of water molecules is inserted at the PP/cellulose interface, then the functionalized cellulose has a higher binding energy with PP. The molecules used to functionalize the cellulose chains are incapable of forming covalent bonds with PP, lowering the interfacial adhesion properties compared to unmodified PP/cellulose composite. Therefore, the silane coupling agents are preferred in the interfacial adhesion research of composite materials due to their capability to covalently bond different components of the composite using hydroxyl and amine groups [34]. Wu et al. [35] improved the interfacial adhesion between cellulose and cementitious paste using the KH550 coupling agent. In their research, they recommended quantitatively evaluating the influence of interfacial adhesion properties on the mechanical performance of cellulose-reinforced cementitious composites. This study covers this research gap by using the classical and reactive MD simulation to characterize the effect of enhanced interfacial adhesion at the silylated cellulose/epoxy interface on the mechanical properties of epoxy composites.

We aim to compare the effects of cellulose content and the silylation treatment on the mechanical properties of epoxy composites. The pull-out simulation is implemented to verify the improvement in interfacial adhesion between cellulose and epoxy, which is achieved by using the KH550 silane coupling agent. The interfacial adhesion at the epoxy/cellulose interface critically affects the ultimate tensile strength of epoxy composites. Therefore, the results of the pull-out simulation should substantially agree with the results obtained from the tensile test, which is simulated using both classical and reactive MD simulations. The epoxy thermoset is reinforced with raw and silylated cellulose at different contents of 28.1–28.3 wt.% and 43.9–44.1 wt.%. The molecular models are prepared for 10 raw cellulose chains ($C_{600}$$O_{500}$$H_{1020}$) and 20 raw cellulose chains ($C_{1200}$$O_{1000}$$H_{2040}$), which are used as reinforcements for unmodified epoxy ($C_{2394}$$N_{152}$$O_{456}$$H_{2964}$) and epoxy functionalized with KH550 (${\mathrm{Si}}_{20}$$C_{2330}$$N_{140}$$O_{500}$$H_{2960}$). The cellulose chains are considered silylated if the nanocovalent bonds are generated between silanol groups of hydrolyzed KH550 and hydroxyl groups of cellulose. The content of this study is classified in four sections. The details of the major elements used to construct the epoxy composites are presented in Section 2, including the introduction of the methods of molecular modeling conducted. Then, the effect of silylation treatment and content of cellulose on enhancing the mechanical properties of epoxy composites is discussed in Section 3. The main outcomes of this paper are summarized in Section 4.

## 2. Materials and Test Methods

Molecular modeling is a practical technique for characterizing the effects of cellulose content and silylation treatment on the interfacial adhesion and, consequently, on the mechanical properties of epoxy/cellulose composites. Further details on the type of ensembles and force fields used for the equilibrium of the molecular systems and the evaluation of force–displacement, energy–displacement, and stress–strain responses are discussed in Section 2.1. We first conduct a pull-out simulation to demonstrate how the hydrolyzed agents of KH550 can enhance the load transfer from epoxy to cellulose reinforcements. Four molecular models are considered for pull-out simulation, representing epoxy/cellulose composites before and after chemical functionalization with the silane agent KH550. The KH550 is used to functionalize the epoxy thermoset and the silylation of cellulose. The details of the epoxy functionalization process and the implementation of silylated cellulose as a reinforcement for epoxy composites are discussed in Section 2.2. The raw and silylated cellulose reinforcements at different weight fractions (28.1–44.1 wt.%) are compared regarding the highest enhancement that can be attained in the maximum values of pulling force, non-bonded interaction energy, ultimate tensile strength, and elastic modulus of the epoxy composites. Then, the force–displacement, energy–displacement, and stress–strain responses for raw cellulose-reinforced epoxy composites are chosen as a baseline to evaluate the enhancements in the interfacial and mechanical properties of their counterparts with silylation treatment.

### 2.1. Molecular Dynamics Simulation

The MD simulations are carried out on a Linux workstation manufactured by MIFCOM GmbH (Munich, Germany), with the following configurations: Intel’s 13th generation core processor (i9-13900K), 64 gigabytes of DDR5 ram, and 2.0 terabytes of SSD disk. The free and open-source software of the large-scale atomic/molecular massively parallel simulator (LAMMPS 2.April.2025) is implemented for MD simulations [36]. LAMMPS is developed by the United States Department of Energy through the Sandia National Laboratories. The MD simulations are implemented to evaluate the force–displacement, energy–displacement, and stress–strain responses of epoxy/cellulose composites, which can be obtained by pulling cellulose out of epoxy composites and by deforming these composites under tensile load, respectively. The pull-out simulations and the characterization of tensile properties are conducted using the polymer consistent force field (PCFF) and its addendum (PCFF+) retrieved from MedeA 3.6 software, Angel Fire, NM, USA [37]. The PCFF and PCFF+ are used to describe the bonded and non-bonded interactions in silylated cellulose and epoxy based on parameters obtained from ab initio calculations. The classical force field of PCFF/PCFF+ contains predefined bonds, angles, and torsions for organic and inorganic materials. However, the PCFF/PCFF+ cannot be implemented to analyze the rupture of nanocovalent bonds among atoms taking place under the tensile load. Hence, reactive force fields (ReaxFFs) are used in this paper to evaluate the ultimate strength of epoxy/cellulose composites. The parameters of ReaxFF describing the atomic interaction between epoxy and cellulose are retrieved from [38,39]. The ReaxFF describes the chemical reactions and the interactions between molecules through evaluating the bond order among atoms as a continuous function of their distance instead of using fixed bonds. This force field offers precise descriptions of bond rupture and bond formation during MD simulations [40].

**Figure 1:**
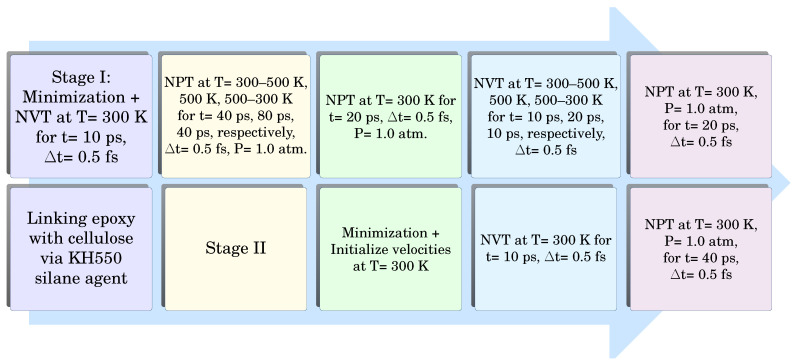
Simulation workflow protocol used for the equilibrium of epoxy/cellulose models related to the tensile test.

For every molecular system considered, six replicates with various initial atomic configurations are generated to enhance the statistical analysis of the evaluated pulling forces, interaction energies, and mechanical properties [41]. Therefore, six separate models of epoxy, raw cellulose, and epoxy/cellulose composites are built and relaxed according to the steps of the equilibrium protocol shown in Figure 1. The recent protocol is applied to relax the molecular models used for the evaluation of the tensile mechanical properties. The equilibrium protocol of Figure 1 is composed of multiple annealing–cooling cycles to eliminate the voids from the molecular models and achieve the maximum possible density. The first annealing–cooling cycle based on the isothermal–isobaric ensemble (NPT) has an equilibrium time of 160 picoseconds, while the second annealing–cooling cycle based on the canonical ensemble (NVT) has a time of 80 picoseconds. Hence, the total equilibrium time is 240 picoseconds, which agrees well with the time used for the equilibrium of similar molecular models relaxed using ReaxFF (200–250 picoseconds) [40,42]. The cellulose molecular models containing up to 10752 atoms can be relaxed even at shorter equilibrium times, which do not exceed 100 picoseconds with a time step size (Δ*t*) = 1.0 femtosecond using the NVT ensemble [43]. The molecular models used for pull-out simulation are relaxed to the final density using NPT ensemble at T = 300 K and P = 1.0 atm for the simulation time of 240 picoseconds with Δ*t* = 0.5 femtoseconds. The molecules are relaxed in the X and Y directions while the cell is fixed in the Z direction. The mechanical properties are increased with increasing density of the molecular systems. The molecular models relaxed with ReaxFF have densities higher than those relaxed with PCFF/PCFF+. Therefore, the tensile stress–strain responses evaluated based on ReaxFF have higher values compared to their counterparts obtained using PCFF/PCFF+.

**Figure 2:**
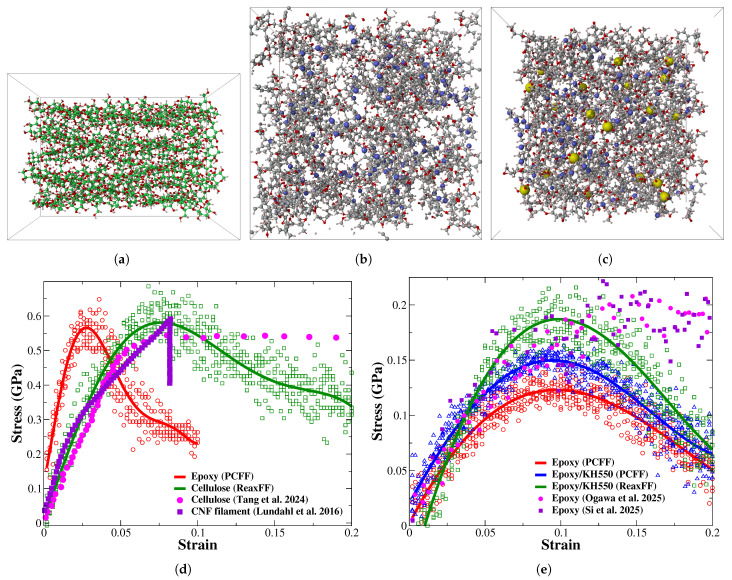
The tensile stress–strain responses of raw cellulose and epoxy evaluated based on PCFF/PCFF+ and ReaxFF forcefields (H: white; O: red; N: blue; Si: yellow; C: green for cellulose and black for epoxy). (**a**) Raw cellulose (20 cellulose chains); (**b**) epoxy; (**c**) epoxy modified with APTES; (**d**) the stress–strain responses of raw cellulose and their corresponding responses retrieved from the literature [52,53]; (**e**) the stress–strain responses of virgin and functionalized epoxy compared to their counterparts in the published literature [48,49].

The tensile modulus and strength are critical strength indicators in a composite material that describe the mechanical behavior of a material under tensile load [44]. The tensile properties of the epoxy/cellulose composites are evaluated based on the Voigt model using the elastic constants matrix [45,46]. The tensile modulus and strength of epoxy and raw cellulose are determined to verify the reliability of ReaxFF and PCFF/PCFF+ in evaluating the mechanical properties of epoxy/cellulose composites. The tensile strength of raw cellulose and epoxy in the axial direction is predicted using the tensile stress–strain responses shown in Figure 2. According to Figure 2d, the ultimate strength values of cellulose are 610 ± 25 MPa and 635 ± 35 MPa, evaluated based on PCFF/PCFF+ and ReaxFF, respectively. These values are close to each other and comparable to the experimental tensile strength of CNF (576 ± 54 MPa) and lyocell fiber (556 ± 78) reported in the literature [2,5,41]. It is noteworthy that the stress–strain responses of cellulose evaluated based on PCFF/PCFF+ and ReaxFF differ in terms of strain values at ultimate tensile strength due to the high proportion of crystalline regions in the molecular models of PCFF/PCFF+ compared to their counterparts of ReaxFF. The yield of cellulose is observed at around 7–8% strain in the molecular models relaxed and deformed using ReaxFF due to the disruption of hydrogen bonds among the cellulose chain segments and a high proportion of amorphous regions [47].

**Figure 3:**
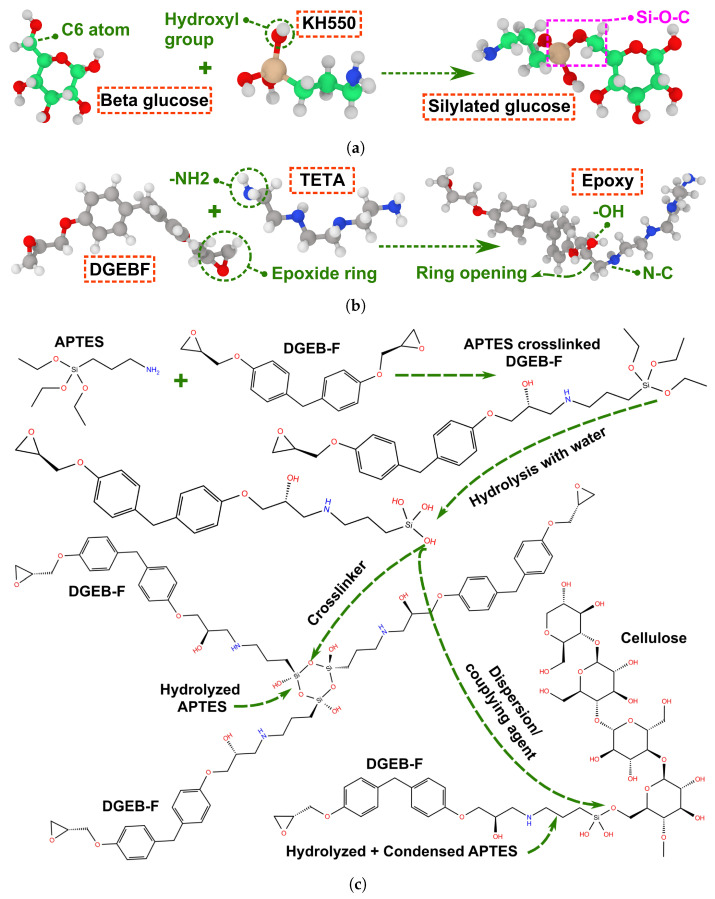
The chemical interactions between the materials considered in the MD simulations. (**a**) The silylation of a glucose unit constituting the cellulose chains; (**b**) the crosslinking of epoxy; (**c**) the functionalization of cellulose and epoxy with hydrolyzed KH550.

According to Figure 2e, the tensile strength values of the epoxy thermoset chemically functionalized with KH550 are 160 ± 3 MPa and 202 ± 15 MPa, predicted based on PCFF/PCFF+ and ReaxFF, respectively. These values are justified for epoxy partially crosslinked with KH550 at 6.6 wt.%. The KH550 molecules improve the structural integrity of epoxy by forming additional bonds via their silanol groups (Si-OH) and amine (${\mathrm{NH}}_{2}$) ends, linking the molecular aggregates of triethylenetetramine (TETA)/diglycidyl ether bisphenol-F (DGEB-F) together as demonstrated in Figure 3c. Furthermore, the tensile strength of epoxy thermoset crosslinked with similar functional groups can reach the value of 190 up to 240 MPa [48,49,50]. The tensile strength of unmodified epoxy is 135 ± 9 MPa, evaluated based on PCFF/PCFF+. Ideally, the theoretical tensile strength values for the vast majority of thermosets are in the range between 200 and 400 MPa. The theoretical strength of epoxy is determined to be 275 MPa, while its maximum true strength value is 166 MPa, evaluated using microtensile tests. The tensile strength of epoxy reported at the micro level is significantly higher than the strength retrieved by the experimental setups of 104 MPa. Therefore, the tensile strength of epoxy can reach its theoretical strength value at small scales, such as micro- and nanolevels [51].

The elasticity of the unmodified epoxy thermoset predicted using PCFF/PCFF+ is 3.39 ± 0.46. The epoxy thermoset modified with KH550 has a close elastic modulus to that of virgin epoxy but a higher tensile strength due to the role of hydrolyzed KH550 molecules in improving its structural integrity. The tensile modulus values of the epoxy thermoset modified with KH550 and raw cellulose evaluated based on PCFF/PCFF+ are 3.53 ± 0.28 GPa and 13.31 ± 1.90 GPa, respectively. The predicted elasticity values of epoxy and cellulose are in satisfactory agreement with previously reported values for epoxy and raw cellulose of 3.3 GPa [54] and 13.5 GPa [55], respectively. Based on the outcomes of the MD simulation implemented to predict the properties of epoxy and raw cellulose, the force fields of PCFF/PCFF+ and ReaxFF can evaluate the tensile properties of these materials with good accuracy. Therefore, PCFF/PCFF+ is used to evaluate the tensile modulus, while ReaxFF is used to evaluate the ultimate tensile strength of the epoxy/cellulose composites. The molecular modeling is carried out using the Schrödinger 2021-2 software developed by D.E. Shaw Research (Manhattan, NY, USA) [56]. The MD simulation studies reviewed in the literature used a 0.60 g/${\mathrm{cm}}^{3}$ value as an adequate starting density for the relaxation of the epoxy- and cellulose-based molecular models to their final densities that are nearly equal to their realistic densities [57,58,59]. Therefore, all molecular models considered in this study have a constant initial density of 0.60 g/${\mathrm{cm}}^{3}$.

The potential energy of the molecular models is minimized using the conjugate gradient method [60]. The force tolerances, force evaluations, maximum iterations, and the initial energy at the first step are $1\times 10^{-4}$, 15,000, 10,000, and 0, respectively. The tensile stress–strain responses are characterized based on the volume-conserving uniaxial strain at 300 K by running nonequilibrium MD simulations based on ReaxFF and PCFF/PCFF+ force fields in the Nose–Hoover NVT thermostat. The simulation cell is elongated in the longitudinal direction in small increments of Δ$\varepsilon_{l}$ = 0.002 at every time step. The time duration for each nonequilibrium NVT ensemble is 5.0 picoseconds with Δ*t* of 0.5 femtoseconds.

### 2.2. Reinforcing Epoxy with Silylated Cellulose

The cellulose considered in this study is not fully amorphous and contains large regions of crystalline cellulose in its structure. Therefore, the molecules of cellulose are designed with a backbone dihedral of 180° and a polymerization degree of 10. Afterwards, the cellulose chains are packed in a way similar to the ones clarified in Figure 2a. The cellulose chain includes amorphous and crystalline regions after applying the equilibrium protocol of Figure 1 to the packed chains. The molecules in the amorphous region are irregularly arranged with large voids, while the molecules in the crystalline region are arranged neatly and tightly. There is no obvious division between the amorphous and crystalline regions in the chain of cellulose, and it is a gradual transition state [43].

**Figure 4:**
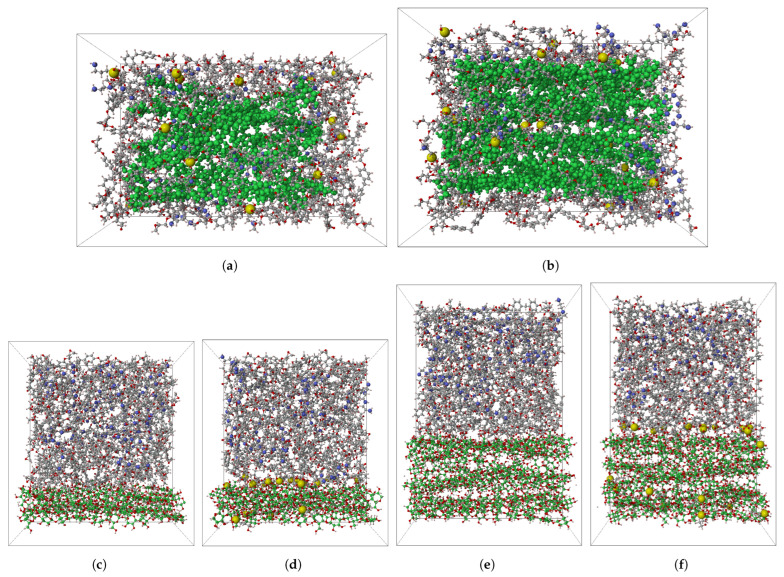
The initial configuration of molecular models used for MD simulations of epoxy/cellulose composites (H: white; O: red; N: blue; Si: yellow; C: green for cellulose and black for epoxy). (**a**) Epoxy-KH550/cellulose (28.1 wt.%); (**b**) epoxy-KH550/cellulose (43.9 wt.%); (**c**) epoxy/cellulose (pull-out) (28.3 wt.%); (**d**) epoxy-KH550/cellulose (pull-out) (28.1 wt.%); (**e**) epoxy/cellulose (pull-out) (44.1 wt.%); (**f**) epoxy-KH550/cellulose (pull-out) (43.9 wt.%).

The silylation treatment is applied on epoxy/cellulose composites using the KH550 silane agent. The KH550 molecules are used as a hardening agent along with TETA to crosslink the monomers of DGEB-F using their amine ends. Then, the crosslinked KH550/TETA/DGEB-F epoxy is hydrolyzed in water to generate highly reactive silanol groups (Si-OH) at KH550 molecules. The silane ends of hydrolyzed KH550 molecules form covalent bonds between each other, improving the interfacial adhesion among the molecular aggregates of TETA/DGEB-F. When the hydrolysis of KH550 is followed by condensation, the hydrolyzed molecules of KH550 in the epoxy matrix act as silane coupling agents, linking cellulose to epoxy through the bonds formed between their silane ends and hydroxyl groups of cellulose. The KH550 molecules are attached to the cellulose through condensation with hydroxyl groups. These molecules can attach to the cellulose substrate through the silane end via Si-O-C nanocovalent bonds or the amine end through ${\mathrm{NH}}_{2}$-OH-C via hydrogen bonds [61]. The dual function of hydrolyzed KH550 as a crosslinker for DGEB-F monomers and as a silane coupling agent for cellulose is clarified in Figure 3c. The open-source software of Packmol v20.2.3 is used to pack the initial molecular models of epoxy/cellulose composites [62]. Figure 4 shows the initial configuration of molecular models used for MD simulations of epoxy/cellulose composites.

The equilibrium protocol of Figure 1 is applied to the molecular models clarified in Figure 4a,b. The protocol includes two stages to eliminate voids from the molecular models, both before and after the crosslinking process. The molecular models are compressed by applying the first stage of the equilibrium protocol. Then, the epoxy matrix is crosslinked with a crosslinking saturation of 50% applied to DGEB-F monomers using the crosslinking algorithm implemented in the Schrödinger 2021-2 software. The crosslinking process of the epoxy matrix is based on the NVT ensemble at T = 800 K and P = 1.0 atm for a total simulation time of 20 picoseconds with Δ*t* = 2.0 femtoseconds. The amine ends of KH550 and TETA are crosslinked with the monomers of DGEB-F according to the reaction clarified in Figure 3b. If the epoxy matrix is reinforced with raw cellulose, then the hydrogen noncovalent bonds are considered at the interface between cellulose and epoxy. However, in epoxy/silylated cellulose composites, the silane coupling agents of hydrolyzed KH550 form covalent bonds with cellulose through their silane ends on one side and connect the silylated cellulose to DGEB-F through their amine ends on the other side. After the crosslinking process is complete for epoxy and cellulose, the second stage of the equilibrium protocol is carried out to eliminate the voids generated by the formation of bonds between molecules.

The ideal weight fraction for raw cellulose-derived fibers used to reinforce epoxy is identified as 40%. The epoxy/cellulose composite exhibits a maximum increase in its mechanical properties at this fiber content [63]. Consequently, the molecular models of epoxy composites are reinforced with two different weight fractions of cellulose at 28.1–28.3% and 43.9–44.1%. The molecular models of epoxy-KH550/cellulose composites shown in Figure 4a,b,d,f are generated by adding 20 molecules of KH550 to 30 molecules of TETA and 110 molecules of DGEB-F. The molecules of epoxy matrix (KH550/TETA/DGEB-F) are used to pack cells containing 10 and 20 cellulose chains. The unmodified epoxy/cellulose composite is obtained by adding 38 molecules of TETA to 114 molecules of DGEB-F, and the resulting epoxy matrix is used to pack 10 and 20 raw cellulose chains shown in Figure 4c and Figure 4e, respectively. The molecules in Figure 4c–f are rearranged to be appropriate for exploring the interfacial adhesion between epoxy and cellulose through the pull-out simulation.

**Figure 5:**
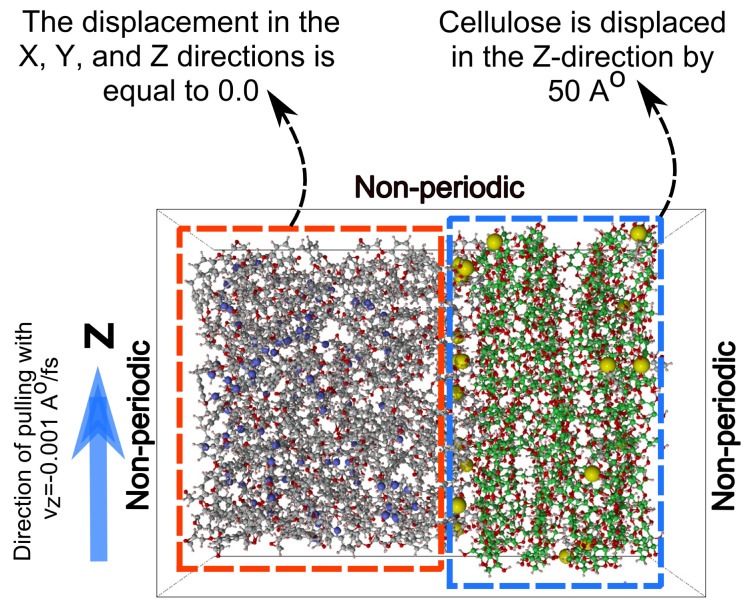
The boundary conditions of velocity and displacement used for the pull-out simulation.

The pull-out simulation is used to verify the function of the KH550 silane coupling agent in improving the interfacial adhesion between epoxy and cellulose. The improvement in the interfacial adhesion is verified through the interaction energy and radial distribution function (RDF) when the cellulose chains are completely separated from epoxy. The RDF describes the probability (g(r)) of locating carbon atoms of epoxy near the reference carbon atoms of cellulose within the specified cutoff radius. The cutoff radius considered in this study for pull-out simulation is set to 12 Å. Figure 5 shows the boundary conditions, including displacement and velocity, used during the pull-out analysis. The steered MD command in the LAMMPS software is used to pull out cellulose from the epoxy composites. The epoxy matrix is fixed in all directions by assigning zero displacement to its molecules. The cellulose chains are moved by 50 Å in the Z-direction with a velocity equal to −0.001 Å/femtosecond. The negative sign is used with the velocity value since LAMMPS considers the cellulose chains as a spring that should be pulled for the achievement of positive displacement in the Z-direction. If a positive sign is used with the velocity value, then LAMMPS will push the cellulose chains in the negative Z-direction. The total number of time steps is 200,000 with Δ*t* = 0.1 femtosecond. The pulling force is recorded for every time step, while the displacement of the cellulose chains is calculated by the subtraction of the current location of the chains at every time step from their initial location. The non-bonded interaction energy between the molecules of epoxy and cellulose is evaluated by applying the compute group/group command of LAMMPS. The bonding energy, which is associated with bonds of epoxy, cellulose, KH550, and the bonds formed among each other, can be predicted by activating only the bonding energy component of the potential energy and then using the commands of compute pe/atom and reduce sum of LAMMPS; the bonding energy in cellulose, epoxy, and KH550 can be obtained.

The exact chemical structure of epoxy, cellulose, and epoxy/cellulose composites, along with the constituents of their matrices and the content of cellulose used to reinforce epoxy, are clarified in Table 1. When the cellulose content is increased from 28.1 to 43.9 wt.%, the loading of KH550 is reduced from 4.8 wt.% to 3.7 wt.%. Specifically, for each 1.0 wt.% of cellulose, 0.17 wt.% of KH550 is available in the epoxy composite reinforced with 28.1 wt.% of cellulose. However, this concentration of KH550 is reduced to 0.08 wt.% for every 1.0 wt.% of cellulose when the content of cellulose is increased from 28.1 wt.% to 43.9 wt.%. Expressing the grafting density change of KH550 in epoxy composites by the language of the molecules’ number, in the epoxy composite with 28.1 wt.% of silylated cellulose, each cellulose chain is attached to two KH550 molecules, but in the composite with 43.9 wt.% of silylated cellulose, each cellulose chain is only attached to one KH550 molecule. Therefore, the silylation treatment in the epoxy composite with 43.9 wt.% of cellulose content yields lower magnitude of improvements in mechanical properties than its counterpart with 28.1 wt.% of cellulose.

## 3. Results and Discussion

The pull-out simulation based on the valence force field of PCFF/PCFF+ is used to verify the enhancement of the interfacial adhesion between cellulose and epoxy due to the implementation of the KH550 silane coupling agent. The MD simulations based on classical and reactive force fields are used to characterize the tensile mechanical properties of epoxy composites reinforced with raw and silylated cellulose. The force–displacement and energy–displacement responses for raw cellulose-reinforced unmodified epoxy are used as a baseline for evaluating the enhancement attained on the interfacial adhesion between epoxy and silylated cellulose. The outcomes of pull-out simulations show that the silylated cellulose-reinforced epoxy at 43.9 wt.% has the highest magnitudes of pulling force, bonding energy, and interaction energy with epoxy thermoset. This conclusion aligns well with the tensile properties characterization results, which indicate that 43.9 wt.% of silylated cellulose provides the highest improvement in the tensile modulus and strength of epoxy composites compared to other cellulose reinforcements. Hence, the silylation of cellulose should be combined with homogeneous dispersion and sufficient loading of cellulose reinforcements (around 40 wt.%) in the epoxy matrix to obtain the highest possible increase in the tensile properties of the resulting composites.

In the following sections, the effects of silylation treatment and cellulose content on the interfacial and mechanical properties of epoxy composites are discussed in detail. Section 3.1 discusses the results of the pull-out simulation that highlight the impact of the KH550 silane coupling agent in improving the interfacial adhesion at the epoxy/cellulose interface. The reinforcing effect of cellulose at different contents on the mechanical properties of epoxy composites is discussed in Section 3.2. Eventually, the impact of silylation on the tensile strength and modulus is analyzed in Section 3.3.

### 3.1. The Effect of Silylation Treatment on the Interfacial Adhesion

**Figure 6:**
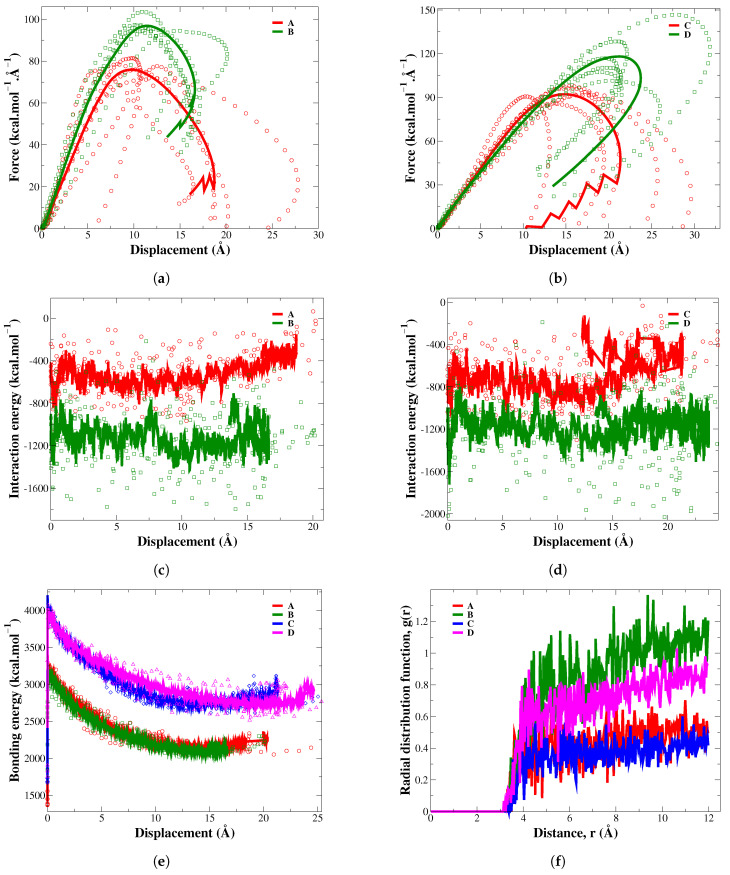
The force and interaction energy responses of epoxy composites reinforced with cellulose at (**a**,**c**) 28.1–28.3 wt.% and (**b**,**d**) 43.9–44.1 wt.%; (**e**) the bonding energy of epoxy composites; (**f**) the RDF of C (cellulose)–C (epoxy); A: epoxy/cellulose (28.3 wt.%); B: epoxy-KH550/cellulose (28.1 wt.%); C: epoxy/cellulose (44.1 wt.%); D: epoxy-KH550/cellulose (43.9 wt.%).

The content of cellulose in the epoxy composites highly affects the effectiveness of silylation treatment in improving interfacial adhesion at the epoxy/cellulose interface. The force–displacement and energy–displacement responses retrieved from the pull-out simulation are shown in Figure 6. The magnitude of force required to pull the cellulose out of the epoxy composite increases with increasing adhesion at the epoxy/cellulose interface. According to Figure 6a, pulling 28.1 wt.% of silylated cellulose out of the epoxy composite requires a force that is 31.08% higher than the force needed to pull 28.3 wt.% of raw cellulose. Increasing the cellulose content from 28.3 wt.% to 44.1 wt.% improves the pulling force by 27.45%. Increasing the cellulose loading in the epoxy composite without applying silylation treatment at the epoxy/cellulose interface is insufficient for increasing the forces required to pull the cellulose. In fact, pulling 28.1 wt.% of silylated cellulose out of the epoxy-KH550 composite requires a force that is marginally higher than the force needed to pull 44.1 wt.% of raw cellulose out of the raw epoxy composite. This conclusion suggests that lightweight epoxy composites reinforced with silylated cellulose can be fabricated by using only half of the raw cellulose content typically used to strengthen unmodified epoxy composites. Increasing the cellulose content in the epoxy composite from 28.3 wt.% to 43.9 wt.%, associated with the application of silylation treatment at the epoxy/cellulose interface, would increase the pulling force by 60%. The results of RDF clarified in Figure 6f indicate that the number of epoxy molecules attached to the 28.1 wt.% of silylated cellulose is higher than those bonded to 43.9 wt.% of silylated cellulose when the cellulose chains are completely pulled out from the epoxy matrix. This behavior can be explained by the ability of each cellulose chain in the epoxy/silylated cellulose (28.1 wt.%) composite to form more nanocovalent bonds with epoxy—specifically, each single cellulose chain can form two nanocovalent bonds with epoxy—compared to its counterpart in the epoxy/silylated cellulose (43.9 wt.%) composite, where a single cellulose chain can only form one nanocovalent bond with epoxy.

Improving the interfacial adhesion between polar cellulose reinforcements and non-polar thermosets, such as epoxy, allows manufacturers of composite materials to use a lower content of cellulose in epoxy/cellulose composites without affecting the mechanical performance of these materials. This observation can be confirmed by the previously published experimental studies that implemented the silylation treatment to improve the interfacial adhesion of cellulose reinforcements with epoxy, consequently increasing the mechanical properties of the resulting composites. Suarsana et al. [64] reinforced epoxy with three different weight fractions of silylated cellulose fibers at 10%, 15%, and 20%. They showed in their experimental study that the epoxy composite reinforced with 20% of silylated cellulose has the highest improvement effect on the flexural strength. Furthermore, the flexural strength of the epoxy composite containing 10 wt.% silylated cellulose and 9% silane agent concentration is 1.5% lower than that of the epoxy composite with 15 wt.% silylated cellulose and 3% silane agent concentration. The epoxy thermoset is reinforced with raw and silylated CNF at two volume fractions of 1% and 1.3%. The epoxy composites reinforced with silylated CNF have a higher tensile modulus and strength compared to their corresponding properties of the untreated epoxy/CNF composites. The tensile modulus of epoxy composite containing raw CNF is approximately equal to that of neat epoxy at the start of deformation and then reduces with increasing strain [65]. The tensile strength of bio-epoxy composite reinforced with 0.9 vol.% of silylated CNF is 19.1% higher than its counterpart of bio-epoxy composite reinforced with 1.4 vol.% of raw CNF [66].

The interaction and bonding energies critically affect the magnitude of the improvement achieved in the pulling force, tensile strength, and modulus of the epoxy composites. The cellulose content and silylation treatment have a direct influence on the values of bonding energy and non-bonded interaction energy, respectively. Referring to Table 2, increasing the content of raw cellulose from 28.3 wt.% to 44.1 wt.% would increase the pulling force by 15.7 kcal·${\mathrm{mol}}^{-1}$·${Å}^{-1}$. This enhancement can be justified by the increase in the interaction and bonding energies of 380.4 and 814.7 kcal/mol, respectively. The KH550 molecules have a small contribution to the total bonding energy according to the values clarified in Table 2. For instance, the difference in bonding energies for epoxy composites reinforced with raw and silylated cellulose are in the range of 22–86 kcal/mol, while the non-bonded interaction energies between cellulose and epoxy are increased considerably by 715.1 up to 839.1 kcal/mol after the application of silylation treatment. The interfacial nanocovalent bonds improve the non-bonded interaction energy between cellulose and epoxy through holding constituents of the epoxy composites tightly together during the deformation process, increasing the resistance of the silylated composites to the pulling force. Therefore, the enhancements attained by using the silane coupling agents of KH550 on the mechanical properties of epoxy composites with identical cellulose contents are mainly attributed to the improved non-bonded interaction energy between cellulose and epoxy. In conclusion, increasing the cellulose content associated with the application of silylation treatment improves the bonded and non-bonded interactions between cellulose and epoxy. The epoxy composite reinforced with 43.9 wt.% of silylated cellulose has the highest magnitude of pulling force, which is attributed to the highest interaction and bonding energies relative to other configurations of epoxy composites listed in the Table 2.

**Figure 7:**
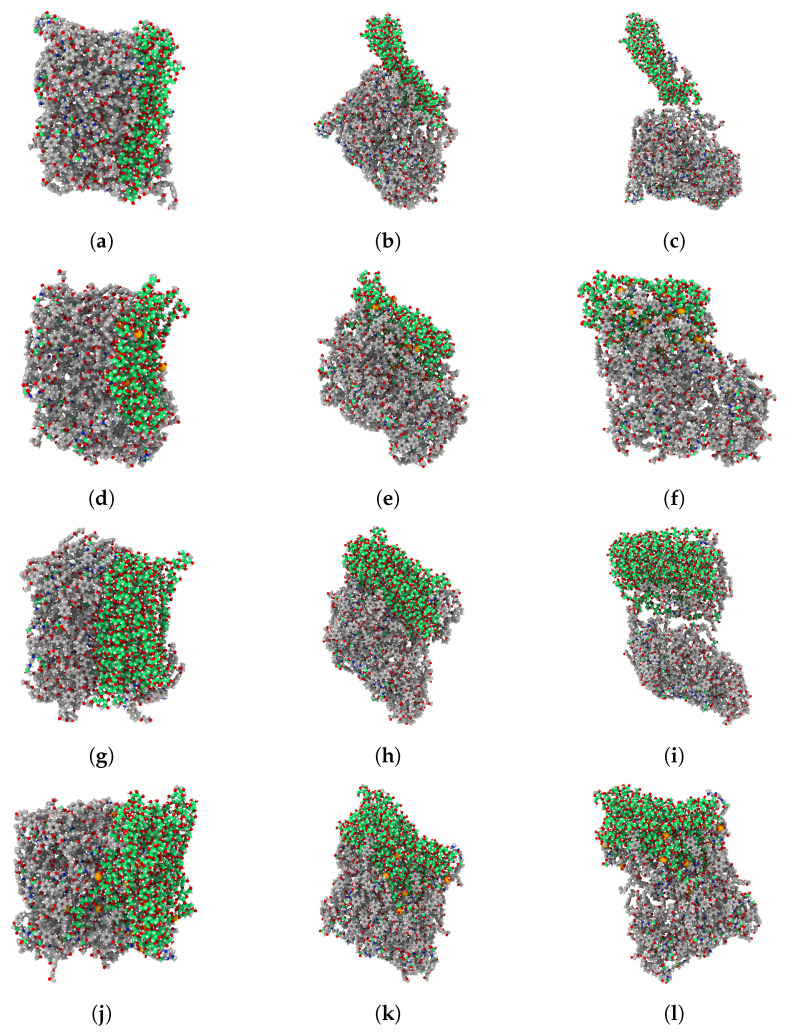
The pulling of cellulose out from epoxy composite. The composites at the first column have zero pulling force; the deformation states of composites at the second column are captured where the maximum pulling force is recorded; the deformation states of composites at the third column are captured at the fracture point. (**a**–**c**) epoxy/cellulose (28.3 wt.%); (**d**–**f**) epoxy-KH550/cellulose (28.1 wt.%); (**g**–**i**) epoxy/cellulose (44.1 wt.%); (**j**–**l**) epoxy-KH550/cellulose (43.9 wt.%).

Figure 7 shows the deformed epoxy composites after pulling the cellulose out of these composites in the Z-direction. The first sub-figure of each row at Figure 7 represent the epoxy composite prior to the application of the pulling force. The unmodified epoxy/cellulose composites have lower resistance to the pulling force compared to their counterparts with silylation treatment. This conclusion can be confirmed from Figure 7b,c. After using KH550 silane as a coupling agent at the epoxy/cellulose interface, the epoxy composites have a higher resistance to the pulling force, as demonstrated in Figure 7e,f. Based on the results of the pull-out simulation, using an epoxy matrix functionalized with KH550 silane coupling agent offers the epoxy/cellulose composites better mechanical performance due to the increased number of bonded and non-bonded interactions at the epoxy/cellulose interface. Therefore, the epoxy matrices are modified with KH550 for all molecular models used for the characterization of tensile mechanical properties under the tensile load. In the following sections, the impact of cellulose loading and silylation treatment is discussed in terms of the improvements achieved in the elastic modulus and tensile strength.

### 3.2. The Structural Reinforcing Effect of Cellulose at High Loadings

**Figure 8:**
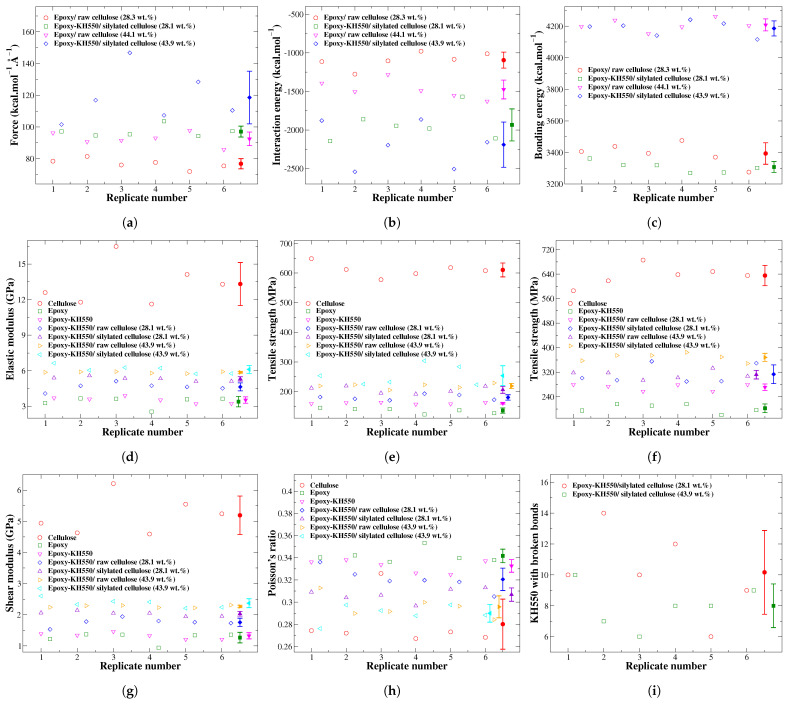
The interfacial and mechanical properties of molecular models’ replicates and their average values are presented. (**a**) Pulling force; (**b**) interaction energy; (**c**) bonding energy; (**d**) elasticity; (**e**) strength (PCFF/PCFF+); (**f**) strength (ReaxFF); (**g**) shear modulus; (**h**) Poisson’s ratio; (**i**) the number of KH550 with ruptured bonds.

The molecular model for each material considered in this study is replicated six times to improve the accuracy of the evaluated interfacial and mechanical properties. The interfacial and mechanical properties of these replicates, along with their averages containing the standard deviation values represented as error bars, are plotted in Figure 8. The pure raw cellulose has the highest mechanical properties relative to other materials, which is used to improve the mechanical performance of the epoxy thermoset. Figure 8e shows that the epoxy thermoset modified with the KH550 silane coupling agent has higher tensile strength in comparison to its counterpart without chemical functionalization due to the capability of the KH550 silane coupling agent to form additional crosslinks between the molecules of the epoxy thermoset.

Referring to Figure 8d,f, the replicates of silylated cellulose-reinforced epoxy composites at 43.9 wt.% have the highest elastic modulus and tensile strength compared to other epoxy composites. This improvement in the strength can be justified by the enhanced interfacial adhesion at the epoxy/cellulose interface, while the tensile modulus is increased due to the increasing content of stiff cellulose in the epoxy matrix. The increasing content of cellulose, along with the application of silylation treatment at the epoxy/cellulose interface, mitigates the Poisson’s ratio values, as demonstrated in Figure 8h, indicating that the epoxy composites become stiffer.

According to the tensile properties listed in Table 3, the cellulose reinforcement has competitive tensile modulus and strength properties of 13.31 ± 1.90 GPa and 635 ± 35 MPa, respectively. These properties are comparable to those of cotton fibers, which have an elastic modulus of 5.5–12.6 GP and a tensile strength of 287–597 MPa [52]. The epoxy thermoset modified with KH550 has an elastic modulus of 3.53 ± 0.28 GPa and tensile strength of 202 ± 15 MPa. The introduction of hydrolyzed KH550 to the chemical structure of epoxy increases its density and improves its tensile strength, while it has a minor effect on its stiffness. The unmodified epoxy resin has an ideal density of 1.14 g/${\mathrm{cm}}^{3}$ and an elastic modulus in the range of 3.30–3.49 GPa [31]. Most of the published studies enhance the mechanical properties of epoxy thermosets by modifying their chemical structure or using reinforcements characterized by superior mechanical performance, such as cellulose. The chemical functionalization of epoxy does not improve all of its material aspects, such as tensile strength, stiffness, toughness, and impact strength. However, modifying the chemical structure of epoxy improves some mechanical properties while reducing others. Furthermore, the chemical functionalization methods of epoxy have an environmental impact that cannot be avoided [67,68,69].

Reinforcing epoxy with cellulose enhances its tensile strength, elastic modulus, and shear modulus. For instance, the raw cellulose at 28.1 wt.% improves the elastic modulus, shear modulus, and tensile strength of epoxy-KH550 by 31.40%, 32.75%, and 33.97%, respectively. Increasing the content of raw cellulose from 28.1 wt.% to 43.9 wt.% would enhance the tensile strength, elastic modulus, and shear modulus of epoxy-KH550 by 54.26%, 66.19%, and 71.16%, respectively. The mechanical properties of epoxy increase as the cellulose content increases if a perfect adhesion at the epoxy/cellulose interface is maintained. These observations have received confirmation from previously published studies. The elastic modulus and ultimate strength properties of epoxy reinforced with 21 wt.% of nanofibrillated cellulose (NFC) are increased by 180.95% and 240.63%, respectively, relative to the properties of neat epoxy. Increasing the content of NFC from 21 to 58 wt.% in epoxy composite would add further enhancements to the ultimate strength and elastic modulus of 48.62% and 66.10%, respectively [70]. The highest improvement in the mechanical properties of epoxy can be attained with a cellulose fiber content of 46 wt.% [19]. The tensile strength values of epoxy composites reinforced with raw cellulose fibers at 44–54 vol.% are in the range between 310 and 320 MPa [2]. These values are in good agreement with the value predicted in this study based on ReaxFF of 312 ± 15 MPa for raw cellulose-reinforced epoxy-KH550 at 43.9 wt.%.

### 3.3. The Effect of Cellulose Silylation on the Mechanical Properties

Increasing the cellulose content beyond 40 wt.% in epoxy composites leads to the deterioration of the interfacial adhesion at the epoxy/cellulose interface [71]. Therefore, we apply the silylation treatment to enhance the dispersion of cellulose reinforcements in the epoxy thermoset, which in turn improves the interfacial adhesion at the epoxy/cellulose interface. The enhanced interfacial adhesion between cellulose and epoxy introduces significant improvements in the tensile strength and modulus of epoxy composites.

**Figure 9:**
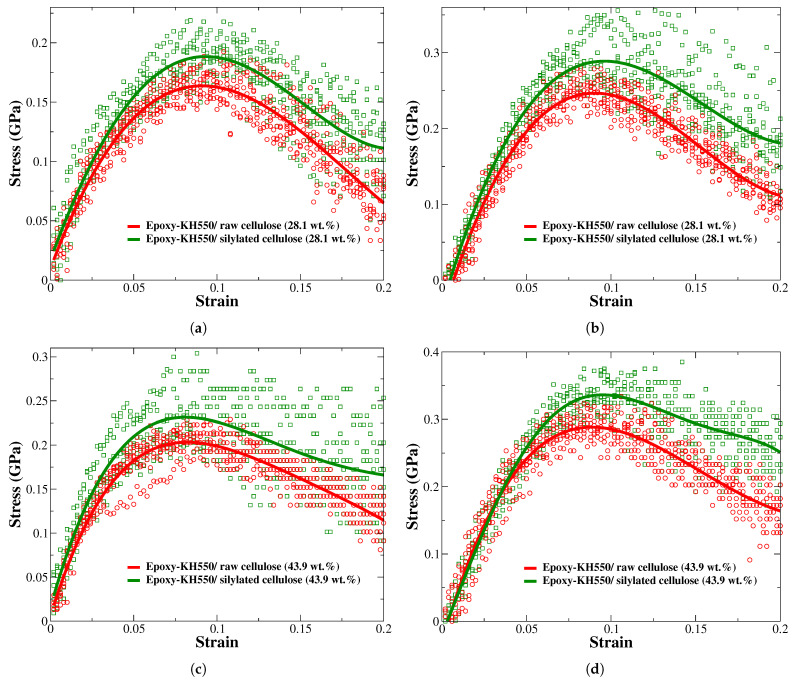
The stress–strain responses retrieved from the tensile test for epoxy/cellulose composites. (**a**) Epoxy-KH550/raw cellulose (28.1 wt.%), PCFF/PCFF+; (**b**) epoxy-KH550/silylated cellulose (28.1 wt.%), ReaxFF; (**c**) epoxy-KH550/raw cellulose (43.9 wt.%), PCFF/PCFF+; (**d**) epoxy-KH550/silylated cellulose (43.9 wt.%), ReaxFF.

Referring to Figure 9, the silylated cellulose at loadings of 28.1 wt.% and 43.9 wt.% improves the ultimate tensile strength of epoxy-KH550 by 54.92% and 82.14%, respectively. The silylation treatment of cellulose has the highest improvement effect on the tensile strength of epoxy-KH550 compared to other properties, such as elastic and shear moduli. The tensile strength of epoxy reinforced with 28.1 wt.% of silylated cellulose (313 ± 32 MPa) is slightly higher than the strength of epoxy composite reinforced with 43.9 wt.% of raw cellulose (312 ± 15 MPa), as demonstrated by the stress–strain responses in Figure 9b,d.

**Figure 10:**
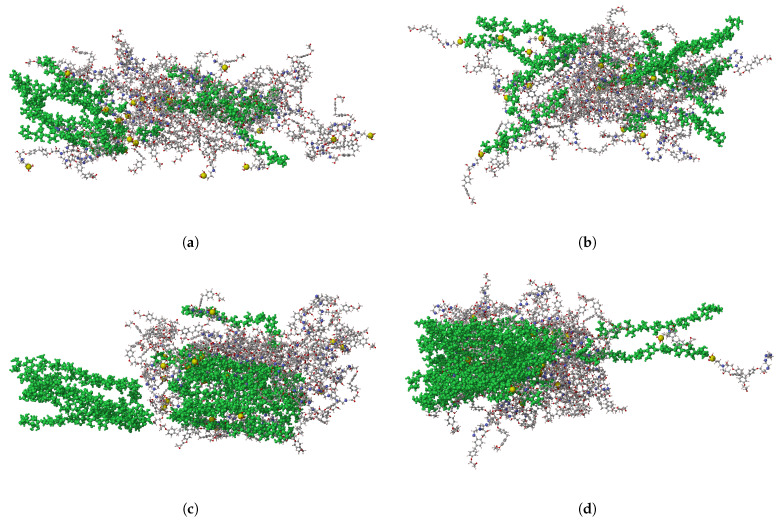
The fracture of epoxy/cellulose composites retrieved from reactive MD simulations. (**a**) Epoxy-KH550/raw cellulose (28.1 wt.%); (**b**) epoxy-KH550/silylated cellulose (28.1 wt.%); (**c**) epoxy-KH550/raw cellulose (43.9 wt.%); (**d**) epoxy-KH550/silylated cellulose (43.9 wt.%).

Figure 10 shows the fractured epoxy/cellulose composites retrieved from reactive MD simulations. The epoxy composite reinforced with raw cellulose at 28.1 wt.% shown in Figure 10a is completely damaged under the effect of the tensile load, while its counterpart reinforced with 28.1 wt.% of silylated cellulose shown in Figure 10b exhibits noticeable resistance to the damaging tensile load. The same conclusion can be drawn from the epoxy composite reinforced with raw cellulose at 43.9 wt.% clarified in Figure 10c and its counterpart reinforced with silylated cellulose at 43.9 wt.% illustrated by Figure 10d. The structural integrity of raw cellulose shown in Figure 10c is highly affected upon the application of the tensile load, indicating that there is a poor load transfer between raw cellulose and epoxy due to weak interfacial adhesion at the epoxy/cellulose interface.

Increasing the content of cellulose reinforcements should be associated with the application of silylation treatment to introduce comprehensive improvements in the tensile properties of epoxy/cellulose composites. The increasing content of cellulose in epoxy/cellulose composites increases their elastic and shear moduli, while the silylation treatment increases their tensile strength due to the improved adhesion at the epoxy/cellulose interface.

## 4. Conclusions

This study compares the impact of cellulose content and silylation treatment on the tensile properties of epoxy composites. We use the hydrolyzed APTES (KH550) as a crosslinker for epoxy and as a silane coupling agent for cellulose. The epoxy composites are reinforced with different contents of cellulose at 28.1–28.3 wt.% and 43.9–44.1 wt.%. The epoxy composites with the epoxy matrix functionalized with KH550 have higher mechanical properties relative to their counterparts fabricated from unmodified epoxy. Therefore, the epoxy matrix partially crosslinked with KH550 is reinforced with raw cellulose. The silylated cellulose reinforcements have covalent bonds with epoxy through the silane and amine parts of the KH550.

The enhancement effect introduced by KH550 on the interfacial adhesion between epoxy and cellulose is investigated by pulling the cellulose out from epoxy composites. The results show that the maximum force required to pull the 28.1 wt.% of silylated cellulose out of the composite is 97 ± 4 kcal·mol·^−1^·Å^−1^, whereas the one needed to pull 44.1 wt.% of raw cellulose is 93 ± 5 kcal·mol·^−1^·Å^−1^. This conclusion is confirmed by the tensile strength values of epoxy/cellulose composites retrieved from reactive MD simulations. The tensile strength of epoxy-KH550/cellulose composite reinforced with 28.1 wt.% of silylated cellulose (313 ± 32 MPa) is higher than its counterpart reinforced with 43.9 wt.% of raw cellulose (312 ± 15 MPa).

The results of MD simulations show that increasing the content of raw cellulose from 28.1 wt.% to 43.9 wt.% would add further improvements to ultimate strength, tensile modulus, and shear modulus by 15.14%, 26.48%, and 28.94%, respectively. The silylated cellulose at 28.1 wt.% enhances the tensile modulus, shear modulus, and strength of epoxy composite by 14.55%, 15.65%, and 15.64%, respectively, compared to its counterpart reinforced with raw cellulose. The application of silylation treatment on the cellulose at 43.9 wt.% increases the elastic modulus, shear modulus, and tensile strength of epoxy/cellulose composite by 4.23%, 4.64%, and 18.07%, respectively. The silylation treatment can effectively increase the tensile strength of epoxy/cellulose composites with the increasing contents of cellulose.

The current study assumes that cellulose reinforcement is uniformly dispersed in the epoxy matrix and that the epoxy thermoset molecules adequately cover the surface of cellulose. All molecular models considered in the analysis are compressed to the maximum possible densities and contain a minor amount of small-sized voids found among the molecules of these models. However, the MD simulations do not consider the effect of large-sized voids, which are generated due to the lack of thermoset molecules and the formation of large cellulose aggregates, on the interfacial and mechanical properties of epoxy composites.

## Figures and Tables

**Table 1:** The molecular specifications of cellulose, epoxy, and epoxy/cellulose composites.

Material	Molecular Formula	Matrix Composition	Cellulose (wt.%)	Number of Atoms
Cellulose	$C_{1200}$ $O_{1000}$ $H_{2040}$	Cellulose chains: 20	-	4240
Epoxy	$C_{2394}$ $N_{152}$ $O_{456}$ $H_{2964}$	Diglycidyl ether bisphenol F molecules: 114, TETA molecules: 38	-	5966
Epoxy/cellulose (28.3 wt.%)	$C_{2994}$ $N_{152}$ $O_{956}$ $H_{3984}$		28.3	8086
Epoxy/cellulose (44.1 wt.%)	$C_{3594}$ $N_{152}$ $O_{1456}$ $H_{5004}$		44.1	10,206
Epoxy-KH550	${\mathrm{Si}}_{20}$ $C_{2330}$ $N_{140}$ $O_{500}$ $H_{2960}$	Diglycidyl ether bisphenol F molecules: 110, TETA molecules: 30, KH550 molecules: 20	-	5950
Epoxy-KH550/cellulose (28.1 wt.%)	${\mathrm{Si}}_{20}$ $C_{2930}$ $N_{140}$ $O_{1000}$ $H_{3980}$		28.1	8050
Epoxy-KH550/cellulose (43.9 wt.%)	${\mathrm{Si}}_{20}$ $C_{3530}$ $N_{140}$ $O_{1500}$ $H_{5000}$		43.9	10,170

**Table 2:** The pulling force, interaction energy, and bonding energy of epoxy composites.

Material	Pulling Force (kcal·${\mathrm{mol}}^{-1}$·${Å}^{-1}$)	Interaction Energy (kcal·${\mathrm{mol}}^{-1}$)	Bonding Energy (kcal·${\mathrm{mol}}^{-1}$)
Epoxy/raw cellulose (28.3 wt.%)	77 ± 3	−1095 ± 109	3393 ± 72
Epoxy-KH550/silylated cellulose (28.1 wt.%)	97 ± 4	−1934 ± 218	3308 ± 36
Epoxy/raw cellulose (44.1 wt.%)	93 ± 5	−1476 ± 128	4208 ± 40
Epoxy-KH550/silylated cellulose (43.9 wt.%)	119 ± 17	−2191 ± 307	4186 ± 50

**Table 3:** Mechanical properties of cellulose, epoxy, and epoxy/cellulose composites.

Material	Elasticity (GPa)	Strength, ReaxFF (MPa)	Strength (MPa)	Shear Modulus (GPa)	Poisson’s Ratio
Cellulose	13.31 ± 1.90	635 ± 35	610 ± 25	5.19 ± 0.65	0.28 ± 0.02
Epoxy-KH550	3.53 ± 0.28	202 ± 15	160 ± 3	1.32 ± 0.10	0.33 ± 0.01
Epoxy-KH550/raw cellulose (28.1 wt.%)	4.64 ± 0.36	271 ± 11	180 ± 10	1.76 ± 0.14	0.32 ± 0.01
Epoxy-KH550/silylated cellulose (28.1 wt.%)	5.31 ± 0.19	313 ± 32	206 ± 13	2.03 ± 0.08	0.31 ± 0.01
Epoxy-KH550/raw cellulose (43.9 wt.%)	5.87 ± 0.08	312 ± 15	219 ± 9	2.27 ± 0.04	0.30 ± 0.01
Epoxy-KH550/silylated cellulose (43.9 wt.%)	6.12 ± 0.36	369 ± 14	254 ± 35	2.37 ± 0.15	0.29 ± 0.01

## Data Availability

The original contributions presented in this study are included in this article. Further inquiries can be directed to the corresponding author.
